# Impression Filler, Surfacer and Separator

**Published:** 1919-08

**Authors:** Rupert E. Hall

**Affiliations:** Chicago, Ill.


### IMPRESSION FILLER, SURFACER AND SEPARATOR

RUPERT E. HALL, D.D.S., CHICAGO, ILL.

Persistent experimentation in the search for a better impression varnish and separating medium resulted in the discovery that the dipping of the impression in collodion produces, we believe, the most perfect and beautifully surfaced impression yet known.

To treat the impression in this manner, secure, as a minimum amount, one pint of flexible collodion (Squibb), and add a little shaving of the lead of an indelible pencil for coloring. Allow the impression to dry for about ten minutes or until the free water has passed away from its surface. Place in the plaster bowl sufficient collodion to permit complete submergence of the impression in it. Dip it into the solution, keeping impression side up. Remove from the solution and stand edgewise to permit excess collodion to drain. In two or three minutes the collodion will dry and the impression will be ready for a second dipping. The first coating fills the impression and makes it impervious to the action of the second. The second acts as a varnish over the first, producing a surface as smooth as that of congealed collodion and approaching that of glass.

The solvent of the collodion, the ether, will attack the impression-tray compound material, so it is desirable to remove the impression from the solution as quickly as its surface has received a coating of the solution.

When finished with the solution, pour it back into the bottle, and cork tightly. As the ether, the vehicle of the solution, is highly volatile, it will gradually evaporate as the solution is used, and the solution will become thickened. When too thick add ether, and thin to the desired consistence.

Cleanse the plaster bowl, after using, before the collodion solidifies; otherwise it will form a hard crust that will destroy the flexibility of the bowl, and when the bowl is forced to bend, the rubber will crack, owing to the adhering unyielding coating of collodion.

Never attempt to paint collodion on the impression with a brush. The ether evaporates so rapidly that it is impossible to apply it uniformly, and it will lap and bridge over the undulations and irregularities of the impression.

Prior to pouring the cast, soak the impression in water to prevent any part of it from absorbing the water from the cast material. Next paint the surface of the impression with soapsuds and rinse off all free soap. The soap will effect easy separation of the impression from the cast. This step is essential, otherwise there is an adhesive tendency between the impression and cast. But be sure to free the surface of the impression of all excess soap by placing the impression under running water, or submerge it in water. Excess soap will produce a defective surface in the cast; the surface will be porous and the quality of the material will be poor. Its action is derogatory to the quality of all cast materials.

The writer's success with the use of collodion, after the manner described, has been so highly satisfactory that he takes pleasure in recommending it as, in his opinion, the one best impression filler and varnisher yet devised. It by far surpasses any other medium and technique with which the writer is familiar, and is a source of satisfaction in his work in daily practice. It gives the finish to a plaster impression, and in turn delivers a smooth, dense cast.—*Dental Cosmos.*

